# Isolation, Identification, Biological Characteristics, and In Vitro and In Vivo Antibacterial Effects of a Bovine-Derived *Escherichia coli* Bacteriophage XJA18

**DOI:** 10.3390/microorganisms14051118

**Published:** 2026-05-14

**Authors:** Zihang Qin, Kaili Guo, Xin Li, Chuanjun Wang, Bao Wang, Rulong Chen, Yunle Cui, Kuojun Cai, Yuefeng Chu, Gang Yao, Xuelian Ma, Yawei Sun, Na Li

**Affiliations:** 1 College of Veterinary Medicine, Xinjiang Agricultural University, Urumqi 830052, China; 320232860@stu.xjau.edu.cn (Z.Q.); 320220061@xjau.edu.cn (X.L.); chenrulong@xjtianlai.com (R.C.); yg@xjau.edu.cn (G.Y.); maxuelian@xjau.edu.cn (X.M.); syw2008@xjau.edu.cn (Y.S.); 2 Xinjiang Key Laboratory of New Drug Research and Development for Herbivorous Animals, Urumqi 830052, China; 3 Center of Diagnosis and Control for Animal Diseases in Bayingolin Mongolian Autonomous Prefecture, Bayingolin 841000, China; 17799707876@163.com; 4 Center of Diagnosis and Control for Animal Diseases in Aletai Prefecture, Aletai 836500, China; 5 Chuangjin Animal Husbandry, Yili 835213, China; 18160566323@163.com (C.W.); wb13394994888@163.com (B.W.); 13150300967@163.com (Y.C.); 6 Center of Diagnosis and Control for Animal Diseases in Urumqi, Urumqi 830092, China; 4642331@163.com; 7 College of Veterinary Medicine, Lanzhou University, Lanzhou 730000, China; chuyf@lzu.edu.cn

**Keywords:** bacteriophage, *Escherichia coli*, phage biological characteristics, whole-genome sequencing, phage therapy

## Abstract

To prevent the spread of antibiotic resistance, bacteriophages have gradually become the most promising alternative to antibiotics for treating bacterial infectious diseases. In this study, using *E. coli* DC1 as the host strain, we isolated a bacteriophage named *Escherichia coli* phage XJA18 from farm sewage. We conducted morphological identification, host range determination, biological characteristic analysis, genomic feature analysis, and evaluation of in vitro and in vivo antibacterial effects. Electron microscopy revealed that phage XJA18 belongs to the class *Caudoviricetes*, with an icosahedral head and a non-contractile long tail. Whole-genome sequencing revealed that the phage has dsDNA with a length of 50,572 bp, with a GC content of 45.33%. The genome does not contain any antibiotic resistance genes or virulence genes, indicating good safety. XJA18 showed lytic activity against 24% of clinically isolated *E. coli* strains. The optimal multiplicity of infection (MOI) was 0.001, with a latent period of 10 min, a burst period of 30 min, and a burst size of 2.22 × 10^2^ PFU/cell. It remained stable at 4–50 °C and pH 4–12. In vitro antibacterial results revealed that XJA18 had the most pronounced initial bacterial growth suppression at MOI = 0.001 during the first 4 h. In vivo experiments demonstrated that both prophylactic and therapeutic administration of XJA18 could protect against *E. coli* infection, significantly reducing inflammatory cytokine levels and bacterial loads in the livers and spleens of mice (*p* < 0.001), significantly increasing body weight (*p* < 0.05), and reducing histopathological damage to the colon, liver, and lungs. In summary, phage XJA18 can effectively inhibit *E. coli* and is safe and stable. These characteristics indicate that phage XJA18 has great potential as a novel biological agent to replace antibiotics for treating bacterial infectious diarrhea in calves.

## 1. Introduction

*Escherichia coli* (*E. coli*) is among the most common causes of calf diarrhea. It affects calf growth performance and the quality and safety of livestock products such as milk [1]. In general, *E. coli* is commonly found in the intestine and is nonpathogenic. However, some *E. coli* strains can acquire specific virulence factors through horizontal gene transfer mechanisms, including bacteriophage transduction, plasmid conjugation, transposon-mediated transfer, and natural transformation, enabling them to cause infectious diarrhea and extraintestinal diseases. These are known as diarrheagenic *Escherichia coli* (DEC) [2,3]. Diarrhea is the leading cause of calf mortality, accounting for more than 50% of total calf deaths. It not only affects the economic benefits of dairy farming but also harms animal welfare [4].

Most cases of calf diarrhea are treated with antimicrobial drugs. A study conducted in the United States revealed that 33.9% of dairy cattle received antimicrobial drugs, and 75% of diarrheal calves received antibacterial drugs [5]. *E. coli*, as a commensal bacterium in the intestines of humans and animals, is in close contact with many other bacteria. It can transfer antibiotic resistance genes to microorganisms that share the same living environment and acquire new resistance genes from them [6]. The proliferation of drug-resistant bacteria has greatly increased the difficulty of treating *E. coli* infections in clinical practice [7]. Long-term and extensive use of antibiotics not only leads to increased drug resistance but also disrupts the balance of normal flora. This promotes the overgrowth of opportunistic pathogens, resulting in diseases that cannot be completely cured and significantly increasing farming costs [8]. Therefore, there is an urgent need to develop green, safe, and effective treatments to address *E. coli* infections in livestock farming, thus promoting healthier animals and safer food. The use of bacteriophages is considered an effective method to combat bacterial multidrug resistance [9].

Bacteriophages are viruses that specifically infect bacteria and archaea. On the basis of their life cycles, they are classified into virulent phages and temperate phages [10]. Virulent phages can rapidly and effectively lyse bacteria through the lytic cycle. They target only specific pathogenic bacteria, exhibit high specificity, do not disrupt normal flora, and are less likely to develop resistance. They are considered natural alternatives to antibiotics [11]. In contrast, temperate phages can integrate their genomes into the host chromosome as prophages and enter a lysogenic state, which may confer new phenotypic traits to the host bacterium via lysogenic conversion. In this study, we focused on virulent phage XJA18 because of its immediate lytic activity and therapeutic potential. Numerous studies have shown that the use of phages to treat intestinal infections caused by pathogenic bacteria such as *E. coli* and *Salmonella* is safe and effective [12,13]. As early as 1987, Smith used 10^5^ PFU/mL of *E. coli* phage to cure diarrheal calves infected with *E. coli*, effectively controlling the proliferation of *E. coli* in the gastrointestinal tracts of calves [14]. In 2021, Alomari used a suppository containing *Lactobacillus* probiotics and phages specific to pathogenic *E. coli* to treat calf diarrhea and achieved good results, indicating that phages have great potential for treating bacterial infectious diseases [15]. Although the application prospects of phages are promising, some challenges need to be overcome, such as the relatively narrow host range of phages, which requires screening for matching phages against clinical isolates [16].

In response to this situation, in this study, a phage, XJA18, was isolated from sewage that lyses calf diarrhea *E. coli*. We analyzed its morphology, biological characteristics, genomic features, and in vitro and in vivo antibacterial effects. This study provides new drug candidates and research evidence for the subsequent clinical use of phages to effectively prevent and control bacterial diarrhea in calves.

## 2. Materials and Methods

### 2.1. Host Strains and Sample Sources

The host strain DC1 and the 25 *E. coli* strains used for host range determination in this experiment were isolated and preserved from clinical calf diarrhea samples at the Key Laboratory of New Drug Research and Development for Herbivorous Animals, College of Animal Medicine, Xinjiang Agricultural University. All the strains were inoculated into LB broth and grown at 37 °C. Sewage was collected from underground well sewage around Urumqi city, Xinjiang, China.

### 2.2. Phage Isolation and Purification

Phage isolation and purification were performed following the method of Sharifull [17]. Five milliliters of collected sewage was mixed with 10 mL of LB broth, and 1 mL of host strain DC1 cultured to logarithmic phase (OD_600nm_ = 0.6) was added. The mixture was incubated overnight at 37 °C with shaking at 180 rpm. After overnight incubation, the culture was centrifuged at 13,400 rpm for 10 min and filtered through a 0.22 µm membrane filter (Biosharp Biotechnology Co., Ltd., Beijing, China) to obtain the phage lysate. Finally, the presence of phages in the culture was determined using the double-layer agar plate method.

Single plaques on the plate were added to 1 mL of liquid medium and incubated at 37 °C for 1 h. The mixture was then centrifuged at 13,400 rpm for 10 min and filtered through a 0.22 µm membrane filter. Plaques were isolated again using the double-layer agar method. This process was repeated five times until plaques of uniform size and clear edges appeared on the plate, indicating that purification was complete. The purified phage stock was stored at 4 °C for subsequent experiments.

The purified XJA18 was diluted tenfold serially, and double-layer agar plates were poured. Each dilution was repeated three times. Plates with 30–300 plaques were selected for counting. The average count was used to calculate the titer.

### 2.3. Electron Microscopy Observation

Following the method of Sharifull, the negative staining method was used to stain the phage, and transmission electron microscopy was used to analyze the phage morphology [17]. Twenty microliters of phage suspension (10^11^ PFU/mL) was dropped onto a copper grid and allowed to stand for 15 min. Excess liquid was absorbed with filter paper. Afterward, 20 µL of 2% phosphotungstic acid was added, and the sample was stained for 5 min. Excess liquid was absorbed with filter paper. After drying, the phage morphology was observed using an HT-7700 Hitachi transmission electron microscope (Hitachi High-Technologies Corporation, Tokyo, Japan) in high contrast mode at 80 kV accelerating voltage and 40,000–80,000× magnification.

### 2.4. Host Range Determination

Following the method of Yue, the spot test was used to determine the host range of XJA18. In total, 25 *E. coli* strains were tested in this study [18]. The test strains were cultured to logarithmic phase. One hundred microliters of the test strain was added to 7 mL of upper-layer LB semisolid medium (0.5% agar content), mixed well, and poured onto lower-layer LB solid medium (1.2% agar content). After the upper layer solidified, 10 µL of phage at a titer of 10^11^ PFU/mL was dropped onto the plate. After drying, the plate was inverted and incubated overnight at 37 °C. The lytic effect of the phage was observed. The appearance of transparent plaques indicated that XJA18 had lytic activity against the test strain.

### 2.5. Optimal Multiplicity of Infection (MOI)

Following the method of Thisara, the viable count method was used to determine the concentration of bacterial culture at the logarithmic phase [19]. Phage XJA18 and the host strain DC1 were diluted and mixed at different concentrations (MOIs = 100, 10, 1, 0.1, 0.01, and 0.001). For all MOI groups, the initial bacterial concentration was standardized at 1.0 × 10^8^ CFU/mL, and phage suspensions were added at 1.0 × 10^6^ to 1.0 × 10^11^ PFU/mL to achieve the respective MOIs. The mixture was incubated at 37 °C with shaking at 180 rpm for 5 h. After incubation, the phage lysate was filtered through a 0.22 µm membrane filter, and the titer was determined using the double-layer plate method. The experiment was repeated three times.

### 2.6. One-Step Growth Curve

Following the methods of Yao, a one-step growth curve was constructed to observe the latent and burst periods of the phage and calculate the burst size [20]. Host strain DC1 and phage were first mixed at the optimal multiplicity of infection (MOI = 0.001) and incubated at 37 °C with shaking at 180 rpm for 15 min. After incubation, the mixture was centrifuged at 13,400 rpm for 5 min. The supernatant was discarded, and the pellet was resuspended in LB broth. The resuspended mixture was centrifuged again at 13,400 rpm for 5 min. This resuspension and washing process was repeated three times to remove free phages. After the final wash, 15 mL of LB broth was added to the pellet, mixed well by shaking, and cultured at 37 °C with shaking at 180 rpm for 120 min. The titer was determined at 0, 5, 10, 20, 30, 40, 50, 60, 80, 100, and 120 min using the double-layer plate method. The experiment was repeated three times.

The burst size was calculated using the formula: Burst size = (P_t − P_0)/B_0, where P_t is the phage titer (PFU/mL) at the end of the burst period (30 min), P_0 is the residual free phage titer after three rounds of centrifugation and washing, and B_0 is the initial bacterial concentration (CFU/mL) at the start of infection. The proportion of infected cells was estimated by comparing the initial bacterial count to the plateau phage count, confirming that >95% of bacteria were infected under the optimal MOI of 0.001.

### 2.7. Thermal and pH Stability

Following the methods of Wang, the thermal and pH stabilities of the phage were determined [21]. Two hundred microliters of phage suspension with an initial titer of 10^8^ PFU/mL was placed in water baths at 4 °C, 20 °C, 30 °C, 40 °C, 50 °C, 60 °C, 70 °C, and 80 °C for 1 h. The phage titer was determined every 30 min using the double-layer plate method. Each temperature and time point was repeated three times. HCl and NaOH were used to adjust the pH of the PBS buffer to 2, 3, 4, 5, 6, 7, 8, 9, 10, 11, and 12. One hundred microliters of phage at a titer of 10^8^ PFU/mL was added to 900 µL of PBS buffer at different pH values, mixed well, and incubated in a shaker (37 °C, 180 r/min) for 1 h. The phage titer was determined using the double-layer plate method. Each pH measurement was repeated three times.

### 2.8. In Vitro Antibacterial Experiments

In vitro antibacterial experiments were conducted following the methods of Zhou et al. [22]. Host strain DC1 and phage were mixed at different concentrations (MOIs = 100, 10, 1, 0.1, 0.01, and 0.001) and added to a 96-well plate. LB broth was used as the positive control (PC) instead of phage, and only LB broth was used as the negative control (NC). Each gradient was set up with three replicate wells. The OD_600_ nm was measured at 0, 1, 2, 3, 4, 5, 6, 7, 8, 9, and 10 h using a microplate reader (BioTek, Winooski, VT, USA).

### 2.9. Whole-Genome Sequencing and Data Processing

Phage genomic DNA was extracted using HiPure Lambda DNA Kits, and the concentration was measured using a NanoDrop 2000 spectrophotometer (Thermo Fisher Scientific, Waltham, MA, USA). After quality inspection, the samples were sent to Benagen Biotechnology Co., Ltd. (Wuhan, China), for whole-genome sequencing. The specific methods were as follows: library construction was performed using the VAHTS Universal Plus DNA Library Prep Kit for Illumina V2 (Nanjing Vazyme Biotech Co., Ltd., Nanjing, China), and whole-genome sequencing was performed using a NovaSeq 6000 after library construction (Illumina, Inc., San Diego, CA, USA).

Raw second-generation sequencing data were filtered using fastp (v2.3.2). Second-generation data were assembled using Unicycler (v0.5.0). Coding genes in the assembled genome were predicted using Prokka (v1.14.6). Functional annotation was performed using HMMER (v3.3.2) on the basis of the Pfam (v35.0) and TIGERfams (v2021-11-10) databases. Antibiotic resistance genes were annotated using RGI software (v6.0.2) on the basis of the CARD antibiotic resistance database. Virulence genes of the phage were annotated using Diamond blastp (v2.0.11) on the basis of the VFDB virulence factor database. Phage genome sequences were compared using NCBI Blast. Collinearity analysis was performed using Mauve software (v2.4.0) to compare the genome structure of phage XJA18 with that of reference phages. A circle map of the genome was constructed using the CGView (v2.1.0) genome visualization tool. A phylogenetic tree was constructed using MEGA 12 with the maximum likelihood method for phage XJA18 and 14 phages after Blast comparison. The bootstrap value was set to 1000 to evaluate the reliability of the phylogenetic tree.

### 2.10. In Vivo Experiments

Thirty female specific-pathogen-free mice (KM mice, 5 weeks old) used for the in vivo experiments were purchased from the Experimental Animal Center of Xinjiang Medical University. The mice were housed in a specific-pathogen-free environment with free access to food and water. The mice were acclimated to this environment for one week before the experiment began. All mouse experiments were approved by the Animal Welfare and Ethics Committee of Xinjiang Agricultural University.

Experimental grouping: Thirty 5-week-old mice were randomly divided into five groups (the control group, phage safety group, infection group, treatment group, and prevention group), with six mice in each group. The mice in the control group were injected intraperitoneally with 0.2 mL of 0.9% normal saline. Mice in the phage safety group (XJA18) were injected intraperitoneally with 0.2 mL of phage XJA18 (10^9^ PFU/mL). Mice in the infection group (DC1), treatment group (Treatment), and prevention group (Prevent) were infected with 0.2 mL of *E. coli* DC1 at a concentration of 1.0 × 10^9^ CFU/mL by intraperitoneal injection. No treatment was given to the infection group after infection. Mice in the treatment group were treated immediately after infection by intraperitoneal injection of 0.2 mL of XJA18 (10^8^ PFU/mL). The mice in the prevention group were given a prophylactic intraperitoneal injection of 0.2 mL of XJA18 (10^8^ PFU/mL) 1 h before infection.

The mice in each experimental group were weighed on days 0 and 7, after which the average daily gain (ADG) was calculated. The mice in the challenge group were euthanized after death, and the surviving mice were euthanized on day 7 of the experiment for sample collection. Whole blood was collected with EDTA anticoagulant for routine blood tests. Serum was used to detect the expression levels of the inflammatory cytokines IL-1beta, IL-6, and TNF-alpha in mice using mouse tumor necrosis factor-alpha (TNF-alpha), mouse interleukin-6 (IL-6), and mouse interleukin-1beta (IL-1beta) enzyme-linked immunosorbent assay kits (Wuhan Elabscience Biotechnology Co., Ltd., Wuhan, China). Sterile liver and spleen samples (0.5 g each) were taken from the mice, homogenized using a magnetic bead grinder, and subjected to plate counting to determine the bacterial loads in the liver and spleen. Heart, liver, lung, kidney, and colon tissues were fixed in 4% paraformaldehyde, embedded in paraffin, sectioned, and stained with hematoxylin and eosin (Besso Biotechnology Co., Ltd., Zhuhai, China). Observations were made under an optical microscope (Leica, Düsseldorf, Germany).

### 2.11. Statistics and Analysis

Statistical analysis and graphing were performed using GraphPad Prism (v10.1.2). Statistical significance was determined using one-way analysis of variance (one-way ANOVA) and the Kruskal–Wallis test, with Tukey correction for multiple comparisons. The results are expressed as the means ± standard deviations (means ± SDs). * indicates *p* < 0.05, indicating a significant difference; ** indicates *p* < 0.01; *** indicates *p* < 0.001, indicating an extremely significant difference.

## 3. Results

### 3.1. Phage Isolation, Purification, and Morphology

**Figure 1:**
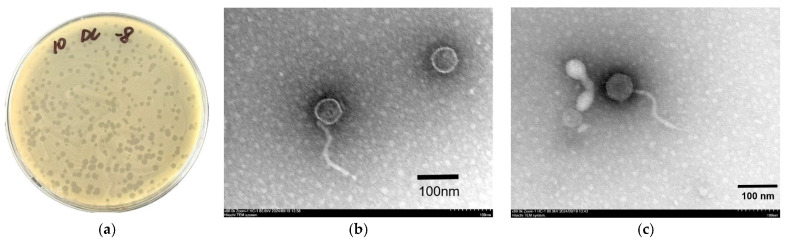
Morphology of phage XJA18 on a double-layer agar plate (**a**) and by transmission electron microscopy (**b**). (**c**) Morphology observed by transmission electron microscopy from another perspective.

Using DC1 as the host strain, the phage XJA18 was isolated. XJA18 formed uniformly sized, clear-edged plaques on double-layer agar plates with a diameter of approximately 1.5–2 mm (Figure 1a). Under transmission electron microscopy, XJA18 was observed to consist of an icosahedral head with a diameter of approximately 67 nm and a non-contractile long tail with a length of approximately 171 nm (Figure 1b,c), belonging to the class *Caudoviricetes*.

### 3.2. Phage Host Range Determination

The host range of phage XJA18 on 25 *E. coli* strains isolated from cattle farms was determined by spot test. The results (Table 1) revealed that 6 strains were lysed, with a lysis rate of 24%. XJA18 showed a lytic ability against *E. coli* strains carrying six virulence genes, namely, csgA (6/6), K88 (2/6), hlyE (5/6), stx2 (2/6), Sta (1/6), F17 (4/6), and eaeA (1/6).

### 3.3. Optimal Multiplicity of Infection (MOI)

The optimal multiplicity of infection determination results revealed that when the MOI was 0.001, the titer of phage XJA18 was the highest, reaching 1.14 × 10^11^ PFU/mL (Table 2). The results indicate that an MOI of 0.001 produces the highest titer.

### 3.4. One-Step Growth Curve

**Figure 2:**
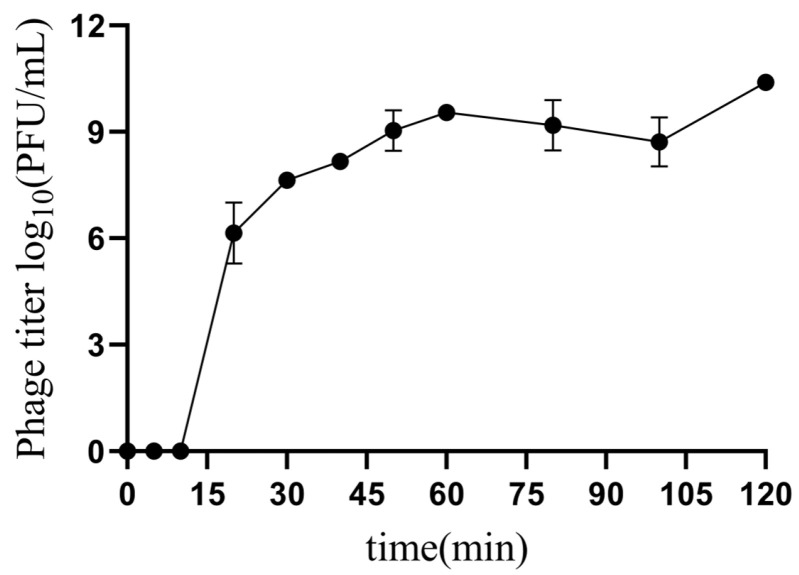
One-step growth curve of phage XJA18.

The one-step growth curve determination results revealed that phage XJA18 proliferated rapidly at 10–20 min, the proliferation rate gradually slowed at 20–30 min, it gradually plateaued after 30 min, and the proliferation rate increased again after 100 min (Figure 2). The latent period of phage XJA18 was 10 min, the burst period was 30 min, and the burst size was 2.22 × 10^2^ PFU/cell.

### 3.5. Thermal and pH Stability

**Figure 3:**
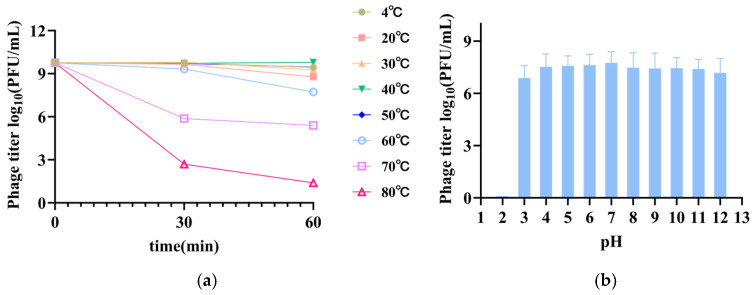
Thermal (**a**) and pH (**b**) stability of phage XJA18.

The thermal stability results revealed that phage XJA18 remained essentially stable when incubated at 4–60 °C for 30 min. The titer decreased by half at 70 °C and decreased significantly at 80 °C. When the samples were incubated at 4–50 °C for 1 h, the titer remained essentially stable. The titer decreased slightly at 60 °C, decreased by half at 70 °C, and was essentially inactivated at 80 °C (Figure 3a). The pH stability results revealed that the phage remained stable at pH 4–12, decreased slightly at pH 3, and was essentially inactivated at pH 2 (Figure 3b). Phage XJA18 has good temperature tolerance and acid–base resistance and can survive stably at 4–50 °C and pH 3–12.

### 3.6. In Vitro Antibacterial Effect

**Figure 4:**
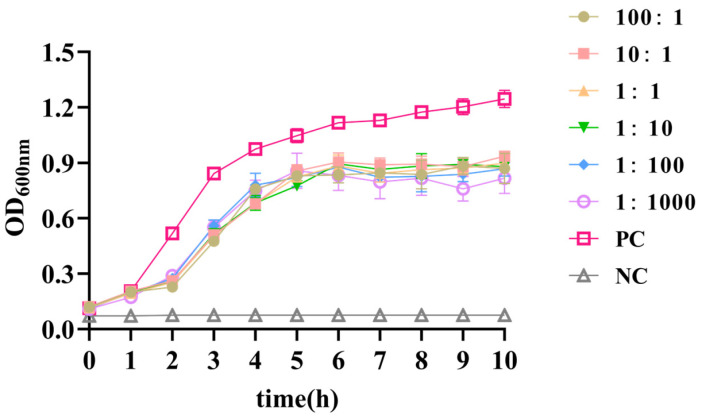
Inhibitory effect of phage XJA18 against *Escherichia coli* in vitro.

The in vitro antibacterial determination results revealed that the OD_600_ of all the groups increased within 4 h but gradually plateaued after 4 h. Compared with that of the group containing only DC1 without XJA18 (PC), the OD_600_ of all the MOI groups decreased, with the lowest OD_600_ occurring at an MOI = 0.001 (Figure 4). This confirms that phage XJA18 causes a rapid reduction in bacterial density within the first 2 h across all tested MOIs, with the most pronounced initial suppression observed at MOI = 0.001, consistent with the optimal propagation MOI. After 4 h, bacterial regrowth was observed in all groups, likely due to the emergence of phage-resistant variants and nutrient depletion.

### 3.7. Whole-Genome Analysis

**Figure 5:**
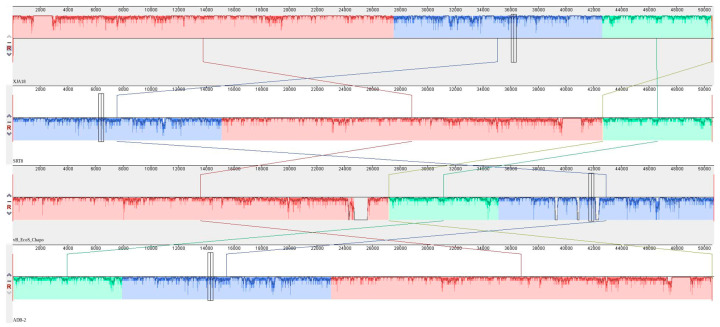
Collinearity analysis of phage XJA18.

Whole-genome sequencing revealed that phage XJA18 is 50,572 bp long, belongs to the class *Caudoviricetes*, has a GC content of 45.33%, and has a circular genome. Blast comparison revealed that the genome sequence of XJA18 has greater than 95% homology with the genomes of the *Escherichia* phage SRT8, the *Escherichia* phage vB_EcoS_Chaps, and the *Escherichia* phage ADB-2, whose genome coverage ranged from 94.21–77.96%. To further compare the genome similarity of these three phages with that of XJA18, collinearity analysis was performed on the four genome sequences (Figure 5).

**Figure 6:**
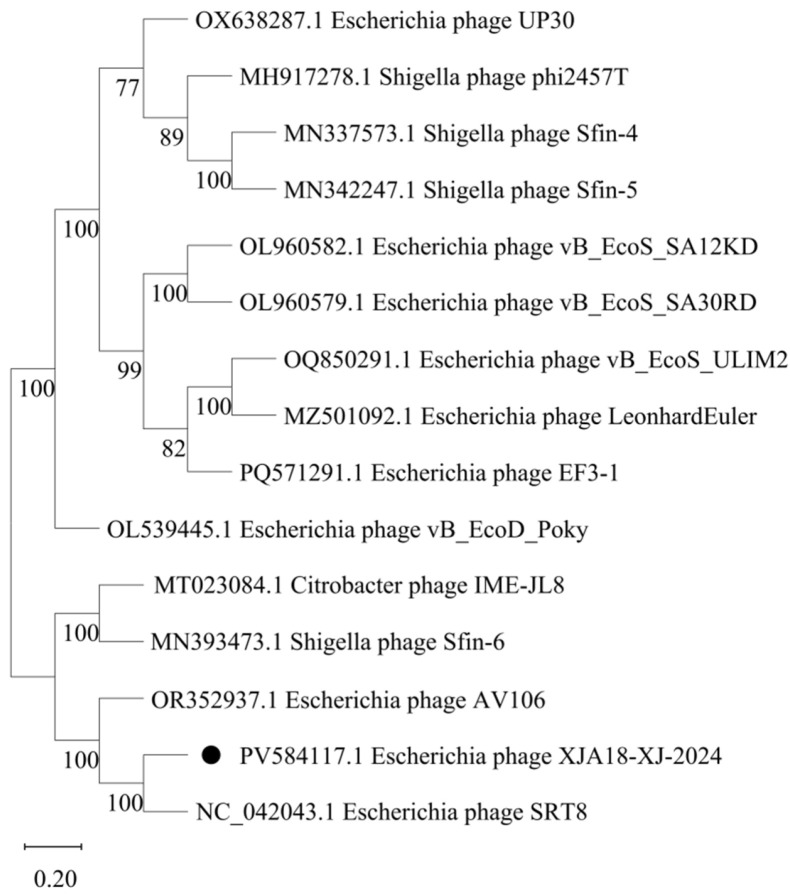
Phylogenetic tree of phage XJA18. Note: The scale bar represents 0.02 substitutions per nucleotide site. The black circle represents phage XJA18.

The results revealed that XJA18 and the *Escherichia* phage SRT8, the *Escherichia* phage vB_EcoS_Chaps, and the *Escherichia* phage ADB-2 are highly homologous at the protein level in most structural regions but show different gene arrangements. A phylogenetic tree was constructed using maximum likelihood for 14 phage genomes with high similarity and the genome of XJA18 (Figure 6).

**Figure 7:**
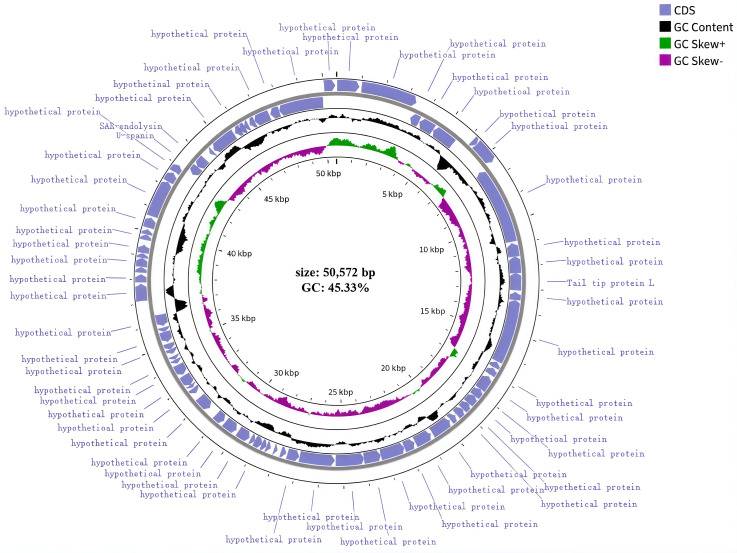
Genomic circular diagram of the bacteriophage XJA18. Note: From outside to inside: first circle, genome coordinates; second circle, genes on the forward strand; third circle, genes on the reverse strand; fourth circle, GC content curve of the genome sequence; fifth circle, GC skew curve of the genome sequence, with purple indicating G < C content.

The results revealed that this phylogenetic tree has two main branches. XJA18 is most closely related to the *Escherichia* phage SRT8 and belongs to the same evolutionary branch. It is more distantly related to the *Escherichia* phage UP30, the *Escherichia* phage vB_EcoD_Poky, the *Escherichia* phage EF3-1qinyuanguan, and the Shigella phage Sfin-5. Comparative genomic analysis with the closest relative, *Escherichia* phage SRT8, enabled the re-annotation of 14 previously hypothetical genes as structural proteins (Figure 7). The genome of phage XJA18 contains 74 predicted coding sequences (CDSs), of which 3 CDSs are functional genes, 2 CDSs are related to phage lysis of Gram-negative bacteria, 1 CDS is a phage structural protein, and the rest are hypothetical proteins (Figure 7). Annotation of antibiotic resistance genes and virulence genes in the CARD and VFDB databases revealed no virulence genes or antibiotic resistance genes, indicating that phage XJA18 is safe for clinical treatment. The genome sequence of phage XJA18 has been deposited in the GenBank database under accession number PV584117.

### 3.8. In Vivo Antibacterial Effect Determination

**Figure 8:**
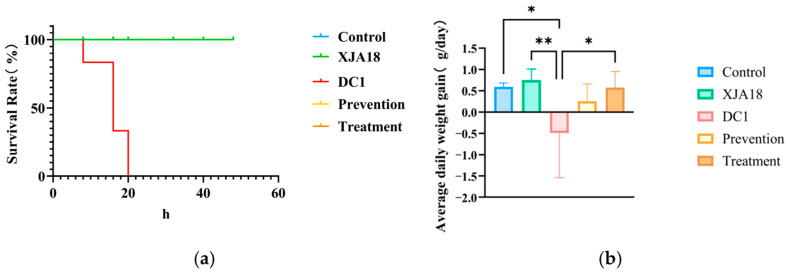
Survival Rate and Average Daily Weight Gain (ADG) of mice across experimental groups. (**a**) Mouse survival curve (**b**) Average daily weight gain of mice. The results are presented as “mean ± standard deviation”, * indicates *p *< 0.05 (significant difference), ** indicates *p* < 0.01.

The safety of phage XJA18, the pathogenicity of host strain DC1, and the prophylactic and therapeutic effects of phage XJA18 on mice infected with DC1 were evaluated in a mouse model. After DC1 infection, the mice in the infection group exhibited trembling and huddling phenomena. All mice in the infection group died within 24 h post-challenge, whereas mice in the control group, phage safety group, treatment group, and prevention group all survived until the end of the 7-day experiment (Figure 8a). The mice in the control group, XJA18 group, treatment group, and prevention group all survived after inoculation. In addition, compared with those in the DC1 group, the body weights of the mice in the XJA18 group increased significantly (*p* < 0.01), whereas the body weights of the control and treatment groups significantly increased (*p* = 0.0168, *p* < 0.05) (Figure 8b). The body weight of the prevention group did not significantly change. These findings indicate that phage XJA18 helps alleviate body weight loss caused by DC1 infection.

**Figure 9:**
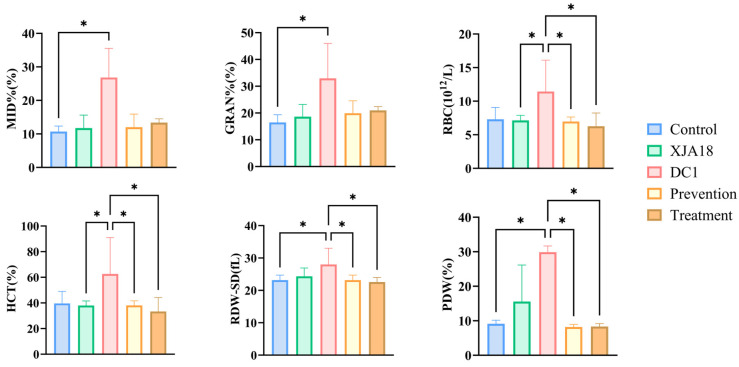
Significantly elevated hematological parameters in challenged mice. The results are presented as “mean ± standard deviation”, * indicates *p *< 0.05 (significant difference).

The results of routine blood tests of the five groups of mice revealed that compared with those in the control and XJA18 groups, the percentage of intermediate cells (MID%), percentage of neutrophils (GRAN%), red blood cell count (RBC), hematocrit (HCT), red blood cell distribution width standard deviation (RDW-SD), and platelet distribution width (PDW) in the DC1 group increased significantly (*p* < 0.05) (Figure 9).

**Figure 10:**
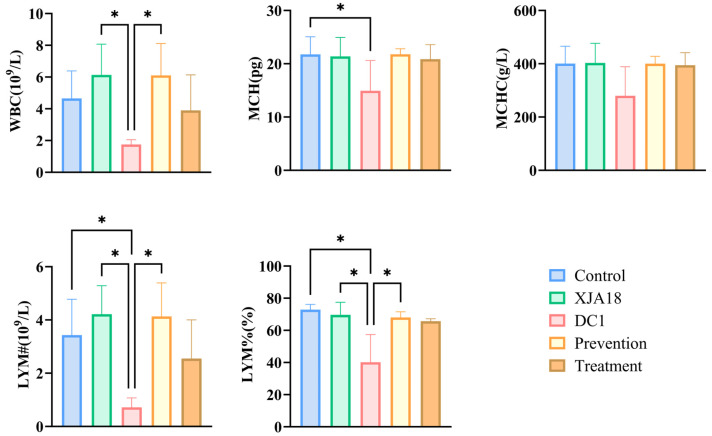
Significantly reduced hematological indices in bacteria-challenged mice.The results are presented as “mean ± standard deviation”, * indicates *p* < 0.05 (significant difference).

The white blood cell count (WBC), mean corpuscular hemoglobin (MCH), lymphocyte count (LYM#), and lymphocyte percentage (LYM%) decreased significantly. Infected mice returned to normal levels after XJA18 intervention (treatment and prevention groups) (*p* < 0.05) (Figure 10). These findings indicate that phage XJA18 has a restorative effect on inflammation, acute infection, and anemia caused by DC1 infection in mice.

**Figure 11:**
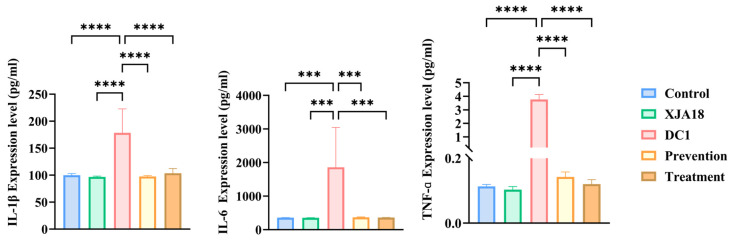
Changes in the levels of the proinflammatory cytokines IL-1β, IL-6, and TNF-α in mice. The results are presented as “mean ± standard deviation”, *** indicates *p* < 0.001, **** indicates *p* < 0.0001.

ELISA results (Figure 11) revealed that the serum levels of the proinflammatory cytokines IL-1beta, IL-6, and TNF-alpha were significantly greater in the DC1 group than in the control and XJA18 groups (*p* < 0.001). After XJA18 intervention (prevention and treatment groups), serum levels decreased significantly (*p* < 0.001), further demonstrating that the phage improved the inflammatory response caused by DC1 infection in mice.

**Figure 12:**
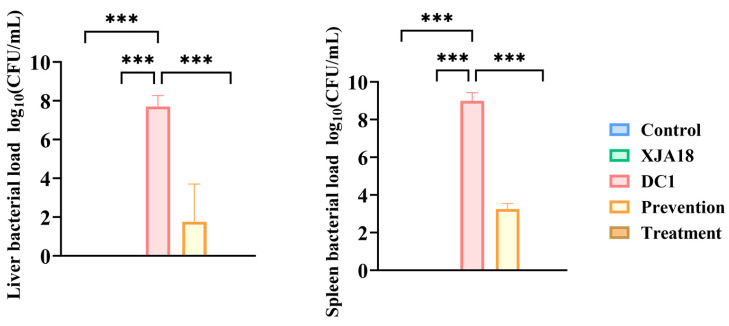
Bacterial load in the mouse liver and spleen. The results are presented as “mean ± standard deviation”, *** indicates *p *< 0.001.

The *E. coli* content in the liver and spleen of the mice was determined using the plate counting method. The results (Figure 12) revealed that the bacterial load in the challenge group was significantly greater than that in the control, phage safety, and treatment groups (*p* < 0.001).

**Figure 13:**
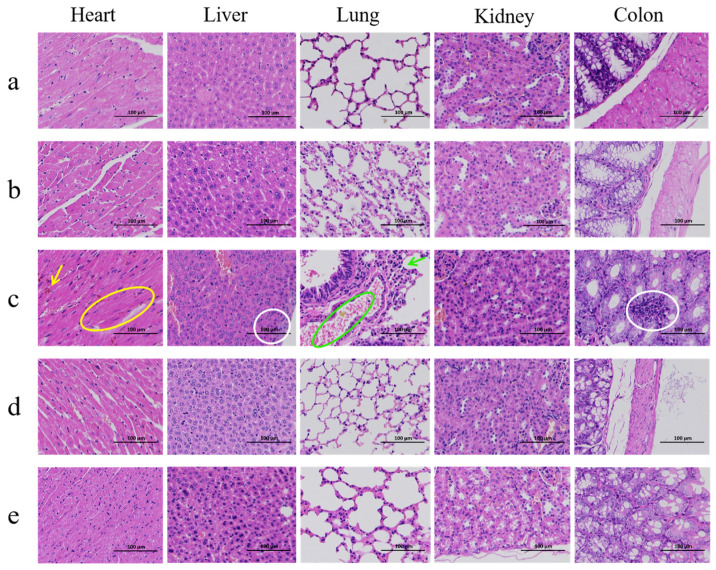
Histopathological sections of mouse tissues. Note: (**a**) Control group, (**b**) XJA18 group, (**c**) DC1 group, (**d**) Prevention group, (**e**) Treatment group. Yellow circles indicate disarray of myocardial fibers, yellow arrows denote interstitial congestion, white circles represent mild inflammatory cell infiltration, green circles indicate alveolar hemorrhage, and green arrows represent thickening of the alveolar wall.

Histopathological observation of the heart, liver, lungs, kidneys, and colon of the mice was performed. The control and phage safety groups contained normal tissue and organs (Figure 13a,b). In the challenge group, obvious pathological damage, including a disordered arrangement of myocardial fibers, interstitial congestion, mild inflammatory cell infiltration in the liver and colon, thickening of the alveolar walls and alveolar hemorrhage in lung tissue, was detected (Figure 13c). The pathological changes in the prevention and treatment groups were significantly reduced, and the tissue structure was essentially restored to near-normal levels (Figure 13d,e).

## 4. Discussion

In this study, a virulent phage, XJA18, was successfully isolated from sewage around Urumqi city, Xinjiang, using highly virulent multidrug-resistant pathogenic *E. coli* DC1 as the host strain. Sewage contains abundant microbial communities and is an important source for phage isolation. This finding is consistent with the fact that phages are widely distributed in natural environments such as water bodies and soil [23,24]. Transmission electron microscopy revealed that XJA18 has typical tailed phage morphological characteristics, with an icosahedral head approximately 67 nm in diameter and a tail length of approximately 171 nm. In accordance with the classification standards of the International Committee on Taxonomy of Viruses (ICTV), this morphological characteristic is consistent with the typical structure of the class *Caudoviricetes* phages [25]. The morphological characteristics of phages are closely related to their infection mechanisms [26]. The long tail structure of the phage suggests that it may specifically recognize lipopolysaccharides, outer membrane proteins, or pili on the surface of the host bacterium through tail fiber proteins, thus injecting genetic material into the host bacterium [27]. Head size is related to genome packaging capacity. The head diameter of XJA18 matches its genome size, which is consistent with the basic law of the relationship between phage structure and function. It is similar in morphology and structure to the phage Xp M29 isolated by Neoralova et al. [28].

The host range determination results revealed that XJA18 has lytic activity against 6 of the 25 tested *E. coli* strains, with a lysis rate of 24%. The lytic spectrum of XJA18 is relatively narrow, which is consistent with the typical high host specificity of virulent phages. Phage host recognition depends mainly on the specific binding of tail fiber proteins to receptors on the surface of the host bacterium [29]. XJA18 showed lytic ability against *E. coli* strains carrying different virulence genes. Its receptor recognition mechanism may target conserved surface structures common to these strains, such as outer membrane protein A or lipopolysaccharide core oligosaccharides, rather than specific virulence factors themselves [30]. Compared with the *E. coli* phage vB_EcoM_FJ1 isolated by Mirzaei, XJA18 has a narrower host spectrum, similar to the phage PECO4 isolated by Zhang et al. [21,31]. This difference may be due to differences in isolation environments and host strains. In this study, clinically pathogenic *E. coli* was used as the host strain, which may have been screened for obligate phages targeting specific pathogenic strains. Environmental isolates usually have a broader host range [32]. Although the narrow host spectrum limits the broad-spectrum application potential of XJA18, it is beneficial for precise treatment and reduces interference with normal intestinal flora [33].

The optimal multiplicity of infection, one-step growth curve, and stability results revealed that phage XJA18 exhibits good biological characteristics. The optimal multiplicity of infection (MOI) of phage XJA18 is 0.001, reflecting the most efficient progeny production under the tested conditions [10]. The one-step growth curve results revealed that the latent period of phage XJA18 was 10 min, the burst period was 30 min, and the burst size was 2.22 × 10^2^ PFU/cell. It has the characteristics of a short latent period and a large burst size, which is very close to the burst size and burst period of the phage vB-EcoS-B2 isolated by Xu, but the latent period is shorter than that of phage vB-EcoS-B2 [18]. These findings indicate that phage XJA18 is associated with rapid infection, a rapid bacterial phagocytosis speed, and strong proliferation ability and can lyse more phages in a shorter time, with potential for clinical application [34]. A short latent period is beneficial for the phage to complete the replication cycle before rapid bacterial proliferation, and a large burst size ensures the effective release and spread of progeny phages [35]. The rapid lytic characteristics of XJA18 may be related to the efficient lytic enzymes encoded in its genome, such as the holin-endolysin system. Whole-genome analysis has confirmed that it contains 2 functional genes related to Gram-negative bacterial lysis [36].

XJA18 activity remained stable at 4–50 °C, with a slight decrease at 60 °C, a 50% decrease in titer at 70 °C, and basic inactivation at 80 °C. In terms of pH stability, XJA18 remained stable at pH 4–12, with decreased activity at pH 3 and inactivation at pH 2. This stability characteristic is similar to that of most tailed phages but better than that of some heat-sensitive phages. Compared with the phage vB_EcoS_ZX4 isolated by Zhang, XJA18 has stronger acid resistance and better gastrointestinal tolerance potential [37,38]. The pH of the rumen of ruminants is usually between 5.5 and 7.0, the abomasum pH can reach 1.5–3.0, and the small intestine pH is in the range of 7.0–8.0 [39]. XJA18 is stable at pH ≥ 4. These results suggest that microencapsulation or liposome encapsulation can be considered in the future to protect the phage through the abomasum and exert antibacterial effects after it reaches the intestine. High-temperature stability allows XJA18 to tolerate conventional cold chain transportation and storage conditions, while a wide acid–base tolerance range is beneficial for its survival in the gastrointestinal environment. In addition, calf feces, urine, and feed residues decompose under the action of microorganisms to produce large amounts of acidic metabolites. The good temperature tolerance and acid–base tolerance of XJA18 allow it to survive well in this environment, serving as an environmental bactericide to eliminate pathogenic bacteria in the environment and reduce the risk of calf diarrhea [40].

Whole-genome analysis is effective for understanding phage characteristics at a deeper level [41]. Genome length reflects the complexity and coding capacity of the phage. GC content is related to genome stability, evolutionary adaptability, gene expression, and regulation. A circular structure is a common form of phage genomes, especially in double-stranded DNA phages [42]. Whole-genome structure analysis revealed that the XJA18 genome is composed of circular double-stranded DNA with a full length of 50,572 bp and a GC content of 45.33%, which is moderate. XJA18 has high homology with the *Escherichia* phage SRT8, vB_EcoS_Chaps, ADB-2, etc. Phylogenetic analysis revealed that XJA18 is most closely related to SRT8 and belongs to the same evolutionary branch. However, there are differences in gene arrangement between XJA18 and the reference phages. This genome rearrangement may be due to recombination events during phage evolution, affecting host range and lytic characteristics [43]. Comparisons with the CARD and VFDB databases revealed that the XJA18 genome does not encode virulence factor genes or antibiotic resistance genes. This “clean genome” characteristic is consistent with the primary criteria for phage therapy proposed by the WHO in 2023, indicating that phage XJA18 is highly safe and meets the standards for clinical phage therapy [44]. Unlike some temperate phages that carry toxin genes or integrase genes, XJA18, as a virulent phage, does not have lysogeny, avoiding the risk of horizontal transfer of virulence genes caused by lysogenic conversion [45]. However, the functional unknownness of hypothetical proteins remains a potential safety concern and can be further analyzed through proteomics and structural biology methods in the future.

Mouse model experiments confirmed the safety and efficacy of XJA18. A safety evaluation revealed that mice inoculated with 10^9^ PFU/mL phage alone showed no clinical symptoms and normal weight gain, indicating that the phage has no acute toxicity to mammals. This is related to the strict host specificity of phages. Phages recognize cell membrane structures unique to prokaryotes and cannot infect eukaryotic cells [46]. Mice in the challenge group had decreased white blood cell (WBC) counts, decreased lymphocyte percentages (LYM%), increased neutrophil percentages (GRAN%), and significantly increased levels of the proinflammatory cytokines IL-1beta, IL-6, and TNF-alpha, indicating typical sepsis characteristics. After phage intervention, the above indicators returned to normal, and the bacterial loads in the liver and spleen decreased significantly, indicating that XJA18 rapidly clears pathogenic bacteria in the circulatory system, blocks the inflammatory cascade, and prevents the occurrence of septic shock [47]. In vivo experiments confirmed the safety of XJA18 in a mouse model, highlighting the potential of phage therapy for preventing and treating *E. coli* infection in mice and providing strong support for further clinical application in the treatment of calf diarrhea. *E. coli* infection may cause systemic acute infection and inflammatory responses in mice, leading to decreased appetite, dehydration, and weight loss [48]. This study revealed that mice infected with DC1 and treated with phage XJA18 had restored body weights and significantly reduced expression levels of proinflammatory cytokines in the body.

In this study, the biological characteristics and safety of phage XJA18 were determined. These results indicate that phage XJA18 has great application potential and is a reliable choice for clinically preventing and controlling calf diarrhea. Phages are being increasingly applied in various fields worldwide [49], such as diarrhea caused by *Salmonella* [50] and *Campylobacter* [51] infections in poultry and mastitis caused by *Staphylococcus aureus* infection in dairy cattle [52]. These findings emphasize the potential of XJA18 not only as a therapeutic drug but also as a preventive intervention in clinical veterinary applications and agricultural production systems. The application of phages in these diseases can not only improve the health and productivity of livestock but also reduce the risk of transmitting antibiotic-resistant bacteria to humans through meat, dairy products, and other animal products [53]. In the future, these successful cases can be used to establish a reasonable local administration plan, using phages as hoof bath solutions for treatment, to better understand the clinical therapeutic effects.

## 5. Conclusions

In this study, the virulent phage XJA18 was isolated from sewage using highly virulent multidrug-resistant *E. coli* DC1 as the host strain. This phage belongs to the class *Caudoviricetes*, with an icosahedral head and a non-contractile long tail, and has a circular double-stranded DNA genome. XJA18 has lytic activity against 6/25 of clinically isolated *E. coli* strains. The optimal MOI is 0.001, the latent period is 10 min, and the burst size is 2.22 × 10^2^ PFU/cell. It is stable at 4–50 °C and pH 4–12. Whole-genome analysis revealed no virulence genes or antibiotic resistance genes, indicating good safety. It showed good prophylactic and therapeutic potential in a mouse infection model and can be used as a single agent or in cocktail formulations to prevent and control calf diarrhea caused by *E. coli* infection in the future.

## Figures and Tables

**Table 1:** Host range of bacteriophage XJA18.

Strain ID	Genus	Source	Carrying Virulence Genes	Carrying Resistance Genes	Lytic Activity
F1-1	*E. coli*	Cattle	*fim*H; *K88*; *hly*E; *stx*2; *F17*; *csg*A	*bla*_SHV_; *bla*_CTX-M_	−
F1-2	*E. coli*	Cattle	*csg*A	*bla*_SHV_; *bla*_CTX-M_	+
F2-1	*E. coli*	Cattle	*F6*; *hly*E; *F17*	*bla*_CTX-M_	−
F2-2	*E. coli*	Cattle	*hly*E; *csg*A	*bla*_SHV_; *bla*_CTX-M_	−
F3	*E. coli*	Cattle	*hly*E; *F17*; *csg*A	*bla*_SHV_; *bla*_TEM_	−
F4	*E. coli*	Cattle	*K88*; *hly*E; *F17*; *csg*A	*bla*_CTX-M_; *bla*_TEM_	−
F5-1	*E. coli*	Cattle	*F6*; *hly*E; *stx*2; *fyu*A; *csg*A	*bla*_CTX-M_; *bla*_TEM_	−
F5-2	*E. coli*	Cattle	*K88*; *hly*E; *hly*A; *csg*A	*bla*_CTX-M_	−
F6	*E. coli*	Cattle	*hly*E; *stx*2; *F17*	*bla*_SHV_; *bla*_CTX-M_	−
F7-1	*E. coli*	Cattle	*Stb*; *hly*E; *F17*; *csg*A	*bla*_SHV_	−
F7-2	*E. coli*	Cattle	*hly*E; *eae*A; *csg*A	*bla*_CTX-M_	−
F8-1	*E. coli*	Cattle	*fim*H; *hly*E; *csg*A	*bla*_CTX-M_; *bla*_TEM_	−
F8-2	*E. coli*	Cattle	*K88*; *hly*E; *stx*2; *csg*A	*bla*_SHV_; *bla*_CTX-M_; *bla*_TEM_	+
F9-1	*E. coli*	Cattle	*Stb*; *hly*E; *csg*A	*bla*_SHV_; *bla*_CTX-M_; *bla*_TEM_	−
F9-2	*E. coli*	Cattle	*Stb*; *hly*E; *stx*2; *F17*; *csg*A	*bla*_SHV_; *bla*_CTX-M_; *bla*_TEM_	−
F10-1	*E. coli*	Cattle	*stx1*; *sta*; *hly*E; *F17*; *csg*A	/	−
F10-2	*E. coli*	Cattle	*Stb*; *hly*E; *csg*A	*bla*_SHV_; *bla*_CTX-M_; *bla*_TEM_	−
F10-3	*E. coli*	Cattle	*Sta*; *K88*; *hly*E; *F17*; *csg*A	*bla*_CTX-M_	+
N6-1	*E. coli*	Cattle	*hly*E; *fyuA*; *csg*A	*bla*_SHV_	−
N6-2	*E. coli*	Cattle	*hly*E; *F17*; *csg*A	*bla*_SHV_	+
N6-3	*E. coli*	Cattle	*Sta*; *hly*E; *eae*A; *csg*A	*bla*_SHV_	−
N7-1	*E. coli*	Cattle	*K88*; *hly*E; *F17*; *csg*A	/	+
N7-2	*E. coli*	Cattle	*stx*1; *hly*E; *eae*A; *csg*A	/	−
N7-3	*E. coli*	Cattle	*F17*; *csg*A	/	−
DC1	*E. coli*	Cattle	*hly*E; *stx*2; *F17*; *eae*A; *csg*A	*bla*_SHV_; *bla*_CTX-M_; *bla*_TEM_; *aac*(6′)-Ib-cr	+

Note: “/” indicates that the four antibiotic resistance genes were not detected in this strain; “+” indicates that phage XJA18 has lytic activity against this strain; “−” indicates that phage XJA18 has no lytic activity against this strain.

**Table 2:** Optimal multiplicity of infection (MOI) of bacteriophage XJA18.

Multiplicity of Infection (MOI)	Initial Bacterial Concentration (CFU/mL)	Initial Phage Titer (PFU/mL)	Post-Culture Phage Titer (PFU/mL)
100:1	1.0 × 10^8^	1.0 × 10^6^	8.8 × 10^10^
10:1	1.0 × 10^8^	1.0 × 10^7^	6.2 × 10^10^
1:1	1.0 × 10^8^	1.0 × 10^8^	1.2 × 10^9^
1:10	1.0 × 10^8^	1.0 × 10^9^	7.7 × 10^10^
1:100	1.0 × 10^8^	1.0 × 10^10^	7.8 × 10^10^
1:1000	1.0 × 10^8^	1.0 × 10^11^	1.1 × 10^11^

Note: The host strain used for all determinations was *E. coli* DC1.

## Data Availability

The original contributions presented in this study are included in the article. Further inquiries can be directed to the corresponding author.
